# Genome-wide assessment of gene-by-smoking interactions in COPD

**DOI:** 10.1038/s41598-018-27463-5

**Published:** 2018-06-18

**Authors:** Boram Park, So-My Koo, Jaehoon An, MoonGyu Lee, Hae Yeon Kang, Dandi Qiao, Michael H. Cho, Joohon Sung, Edwin K. Silverman, Hyeon-Jong Yang, Sungho Won

**Affiliations:** 1 Department of public health sciences, Seoul national university, Seoul, Korea; 2 Division of Allergy and Respiratory Medicine, Department of Internal Medicine, Soonchunhyang University Seoul Hospital, Soonchunhyang University College of Medicine, Seoul, Korea; 3 SCH Biomedical Informatics Research Unit, Soonchunhyang University Seoul Hospital, Seoul, Korea; 4 Department of Internal Medicine, Healthcare Research Institute, Seoul National University Hospital Healthcare System Gangnam Center, Seoul, South Korea; 5 Channing Division of Network Medicine, Department of Medicine, Brigham and Women’s Hospital and Harvard Medical School, Boston, Massachusetts, United States of America; 6 Division of Pulmonary and Critical Care Medicine, Department of Medicine, Brigham and Women’s Hospital, Harvard Medical School, Boston, Massachusetts, United States of America; 7 Interdisciplinary Program of Bioinformatics, Seoul National University, Seoul, Korea; 8 Institute of Health and Environment, Seoul National University, Seoul, Korea; 9 Pediatric Allergy and Respiratory Center, Department of Pediatrics, Soonchunhyang University Seoul Hosplital, Soonchunhyang University College of Medicine, Seoul, Korea

## Abstract

Cigarette smoke exposure is a major risk factor in chronic obstructive pulmonary disease (COPD) and its interactions with genetic variants could affect lung function. However, few gene-smoking interactions have been reported. In this report, we evaluated the effects of gene-smoking interactions on lung function using Korea Associated Resource (KARE) data with the spirometric variables—forced expiratory volume in 1 s (FEV_1_). We found that variations in FEV_1_ were different among smoking status. Thus, we considered a linear mixed model for association analysis under heteroscedasticity according to smoking status. We found a previously identified locus near *SOX9* on chromosome 17 to be the most significant based on a joint test of the main and interaction effects of smoking. Smoking interactions were replicated with Gene-Environment of Interaction and phenotype (GENIE), Multi-Ethnic Study of Atherosclerosis-Lung (MESA-Lung), and COPDGene studies. We found that individuals with minor alleles, rs17765644, rs17178251, rs11870732, and rs4793541, tended to have lower FEV_1_ values, and lung function decreased much faster with age for smokers. There have been very few reports to replicate a common variant gene-smoking interaction, and our results revealed that statistical models for gene-smoking interaction analyses should be carefully selected.

## Introduction

The spirometric measurement forced expiratory volume in the first second (FEV_1_) reflects the physiological and functional state of the lungs; this measure has been used as the gold standard for diagnosing a lung disease, classifying its severity, assessing its progression over time, and monitoring the treatment response^1^. Furthermore, this parameter is a predictor of other morbidities and mortality in the general population, even independent of smoking history^2,3^. Reduced FEV_1_ is a characteristic of chronic obstructive pulmonary disease (COPD), a leading cause of mortality and morbidity worldwide^4,5^. The prevalence and burden of COPD are expected to increase in the coming decades owing to continued exposure to COPD risk factors and the aging population^5^.

Multiple risk factors for COPD have been identified, and smoking has been recognized as the major risk factor for a rapid decline in lung function and consequent development of COPD. However, only a minority of smokers develop COPD^6^, and there are substantial differences in the sensitivity to smoking among individuals. These differences are partly attributable to genes and/or their interactions with smoking. Heritability estimates of lung function range from 39% to 66%^7,8^. Moreover, a hereditary severe deficiency in alpha-1 antitrypsin, encoded by *SERPINA1* on chromosome 14^9^, is the best known genetic risk factor for the development of COPD. However, severe alpha-1 antitrypsin deficiency accounts for only about 1% of the patients with COPD^10^. Thus, improving our understanding about disease pathogenesis and progression would require studies on genetic susceptibility loci and their interactions with smoking.

Recently, many genome-wide association studies (GWASs) have been conducted to identify the genetic loci associated with lung function levels, and many genome-wide significant loci have been identified. The necessity of replication across populations with diverse ethnic or environmental characteristics has been reported^11^. However, loci identified from GWASs have often failed to be replicated in different populations^12^. There are many reasons for this inconsistency, and several studies have shown a partial relation with gene-environment interactions^11,13^. Smoking has a strong effect on lung function, and the effects of gene-smoking interactions on lung function have been repeatedly highlighted^14–16^. However, the effects of gene-smoking interactions on variability in lung function in different ethnicities are not clear. Furthermore, because COPD is expected to be the fifth most common disease worldwide, with the third highest mortality rate^5^ in 2020, and because its burden, including financial cost, is predicted to increase, early prediction of lung function may be important for developing individualized therapeutic strategies, and further studies are required to identify genetic factors that predict the risk for a subsequent rapid decline in lung function across ethnicities.

In this study, we aimed to identify the genetic variants interacting with smoking on FEV_1_ using genome-wide interaction studies (GWISs). We considered various models in terms of smoking-related variables and variance-covariance structure, and the best model was chosen by Akaike information criterion (AIC) for each dataset. GWISs were conducted using the Korea Associated Resource (KARE) data. We detected that SNPs with the smallest P-values are located near *SOX9*. The *SOX9* has been reported to be involved in lung branching morphogenesis^17^ and recovery of lung function after lung injury^18^. It should be noted that genetic association of *SOX9* was firstly detected by Hankcock *et al*., but their interaction P-values were larger than 0.05^19^. We replicated interactions in the Gene-Environment of Interaction and phenotype (GENIE), Multi-Ethnic Study of Atherosclerosis-Lung (MESA-Lung), and COPDGene studies. Our findings provide important insights into our understanding about lung disease prevention and control.

## Methods

### Data description

Our analyses consisted of two phases—discovery and replication. For the discovery phase, we conducted GWISs on FEV_1_ using KARE data. For the replication phase, we considered GENIE, MESA-Lung, and COPDGene data, and replicated the significant results identified from the filtering step. Detailed procedure for genotyping, quality controls (QC), and imputations for each data are described in the Supplementary Text 1.

#### KARE

Data collected by the KARE project were used for GWASs. Participants were recruited from the rural Ansung and urban Ansan cohorts. Initiated in 2001 as part of the Korean Genome Epidemiology Study (KoGES), the initial samples included 5,018 and 5,020 participants aged 40–69 years from Ansung and Ansan areas, respectively. After QC of genotypes, there were 8,534 participants between the age of 40 and 69 years with at least one spirometry test and genotype data (see Supplementary Text 1 for detailed procedures about QC). Among these participants, 4,001 were men and 4,533 were women. The values of FEV_1_ were observed up to three times every two years, and 19,557 measurements were used for the analyses. Smoking history was obtained through a questionnaire, and smoking status and pack years were used for association analyses as covariates. Smoking status was categorized as never smokers, former smokers, and current smokers. Never smokers were defined as individuals who had never smoked, and former smokers were participants who had smoked previously, but stopped smoking prior to the survey. Current smokers were individuals who stated that they currently smoked during the investigation, or who had a record of smoking and did not belong to the other two categories. According to our categorization, there were 4,926 never smokers, 1,742 former smokers, and 1,866 current smokers in our cohort.

#### GENIE

GENIE data were used to replicate SNPs identified from GWISs using KARE data. The GENIE cohort consisted of 7,999 participants, who had visited Seoul National University Gangnam Center during 2014^20^. They agreed to participate in genetic studies and donated blood samples, and after QC, there were 5,971 participants (3,404 men and 2,567 women) with spirometry and smoking-related variables (see Supplementary Text 1 for detailed procedures about QC). Spirometry and smoking-related variables were repeatedly measured up to 11 times. Smoking-related variables were obtained by questionnaire. Based on their responses, smoking status was categorized into three groups, similar to the smoking status categories used in the KARE data. The numbers of never smokers, former smokers, and current smokers were 3,396, 1,804, and 771, respectively.

#### MESA-Lung non-Hispanic whites

MESA-Lung data were used to replicate SNPs identified from GWISs using KARE data. MESA was a prospective cohort initiated to investigate cardiovascular diseases. Participants consisted of 6,814 men and women aged 45–84 years. As a subgroup of the MESA cohort, the MESA-Lung study enrolled 3,965 participants who were sampled from the MESA cohort, and agreed to participate in the genetic analysis and to measure their lung functions^21^. The MESA-Lung study was composed of four populations—non-Hispanic whites (NHWs; 35%), African-Americans (AAs; 26%), Hispanics (23%), and Chinese-Americans (16%)^21^—and we considered only NHWs. After QC of genotypes, 1,033 participants had both spirometry and smoking-related variables with genotype data, including 459 never smokers, 468 former smokers, and 106 current smokers (see Supplementary Text 1 for detailed procedures about QC).

#### COPDGene

COPDGene was a multi-center study on smokers with and without COPD, and included AAs and NHWs. All participants had at least 10 pack years of smoking, and their ages were between 45 and 80 years. Pre- and post-bronchodilator spirometric data were obtained for all participants with standardized spirometry (EasyOne Spirometer; Zurich, Switzerland); to be consistent with other studies, we focused on pre-bronchodilator spirometry. After QC of genotypes, 7,760 NHWs and 3,300 AAs were enrolled for replication studies^22^ (see Supplementary Text 1 for detailed procedures about QC). AAs consisted of 2,643 current smokers and 657 former smokers, and NHWs consist of 2,616 current smokers and 4,054 former smokers.

### Statistical analysis

Smoking has a significant effect on respiratory function, and it was found that variances of FEV_1_ can differ by smoking status. Linear mixed models which allow heteroscedasticity according to smoking status were computationally very intensive and we consider a two step approach; (i) filtering step and (ii) testing step. In the filtering step, participants were stratified according to the smoking status and a likelihood ratio test with 3 degrees of freedom which allow heteroscedasticity according to smoking status were applied to select the most significantly associated SNPs. Filtering step was considered only for discovery data, and the likelihood ratio test will be called 3 DF test in the remainder of this article. For testing step, we consider several variance-covariance structures for the linear mixed models, and the best model was chosen with AIC. Then former, current, and never smokers were pooled, and linear mixed models with the smallest AIC were applied to the SNPs selected from the filtering step.

#### Filtering step: genome- wide interaction studies (GWISs) with KARE data

We considered FEV_1_ (mL) as spirometric measures, which were used to identify the genetic variants interacting with smoking. GWISs were conducted using KARE data. We found that there were no substantial differences in spirometric measures between current smokers and former smokers for KARE data with AIC, and both groups were combined into a single group; ever smokers. To handle heteroscedasticity, we conducted stratified analyses which applied linear mixed models to never smokers and ever smokers separately for GWISs. For both groups, sex, age at baseline measurement, height, body mass index (BMI), elapsed time from the baseline measurement, and interaction of age and sex were included as covariates. It was reported that lung function decline accelerates after he or she becomes 35 years old^23^, and all participants in KARE data were around 40–69 years old at the baseline. Thus, effect of ages on the baseline pulmonary function measurements and that of elapsed time on their decrement were expected to be substantially different, and it is a main reason why the baseline age and elapsed time from the baseline measurement were considered as different covariates. To adjust for population substructure strictly, principal component (PC) analyses were applied to the genetic relationship matrix, and the first 10 PC scores were included as covariates^24^. FEV_1_ of each participant was measured up to 3 times, and FEV_1_ at each time point was included as response variables. The similarities among repeated spirometric measures for each participant were handled with a random intercept. We let *y*_*ij*_ be FEV_1_ values for participant *i* at time point *j*, and they were assumed to follow multivariate normal (*MVN*) distribution. We denote elapsed time from baseline measurement and PC scores for participant *i* and component *k* by time_*ij*_ and pc_*i*_^*k*^. Then the linear mixed model for ever smokers becomes

1 $$\begin{array}{rcl}{y}_{ij} & = & {\beta }_{0}+{\beta }_{1}{{\rm{age}}}_{i}+{\beta }_{2}{{\rm{sex}}}_{i}+{\beta }_{3}{{\rm{BMI}}}_{ij}+{\beta }_{4}{{\rm{height}}}_{ij}\\  &  & +\,{\beta }_{5}{{\rm{time}}}_{ij}+{\beta }_{6}{\rm{pack}}\,{{\rm{year}}}_{ij}+{\beta }_{7}{{\rm{sex}}}_{i}\,\cdot \,{{\rm{age}}}_{i}+{\beta }_{8}{{\rm{SNP}}}_{i}+{\beta }_{9}{\rm{SNP}}\,\cdot \,{\rm{pack}}\,{{\rm{year}}}_{ij}\\  &  & +\,\sum _{k=1}^{10}{\tau }_{k}{{\rm{pc}}}_{i}^{k}+{b}_{i}+{\varepsilon }_{ij}\,,\,{({\varepsilon }_{ij1},\mathrm{...},{\varepsilon }_{i{n}_{i}})}^{t} \sim MVN\,(0,{\rm{\Sigma }}),\,{b}_{i} \sim iid\,MVN\,(0,{\sigma }^{2})\end{array}$$

For never smokers, we consider

2 $$\begin{array}{rcl}{y}_{ij} & = & {\beta ^{\prime} }_{0}+{\beta ^{\prime} }_{1}{{\rm{age}}}_{i}+{\beta ^{\prime} }_{2}{{\rm{sex}}}_{i}+{\beta ^{\prime} }_{3}{{\rm{BMI}}}_{ij}+{\beta ^{\prime} }_{4}{{\rm{height}}}_{ij}\\  &  & +\,{\beta ^{\prime} }_{5}{{\rm{time}}}_{ij}+{\beta ^{\prime} }_{6}{{\rm{sex}}}_{i}\cdot {{\rm{age}}}_{i}+{\beta ^{\prime} }_{7}{{\rm{SNP}}}_{i}+\sum _{k=1}^{10}{\tau }_{k}{{\rm{pc}}}_{i}^{k}\\  &  & +\,{b^{\prime} }_{i}+{\varepsilon ^{\prime} }_{ij}\,,\,\,{({\varepsilon ^{\prime} }_{ij1},\mathrm{...},{\varepsilon ^{\prime} }_{i{n}_{i}})}^{t} \sim MVN\,(0,{\rm{\Sigma }}^{\prime} ),\,{b^{\prime} }_{i} \sim iid\,MVN\,(0,{\sigma ^{\prime} }^{2})\end{array}$$

It should be noted that pack years are 0 for never smokers and were included as covariates only for ever smokers. We compared several structures for Σ and Σ′, and selected an unstructured covariance structure. The proposed models were applied to detect gene-smoking interaction of FEV_1_ average levels. To identify SNPs interacting with smoking on spirometric measures, we considered H_0_: ${\beta ^{\prime} }_{7}={\beta }_{8}={\beta }_{9}=0.$ This could be tested by summing a likelihood ratio test with 2 degrees of freedom (DF) for ever smokers and a likelihood ratio test with 1 DF for never smokers. The summed likelihood ratio test followed the chi-square test with 3 DF under the null hypothesis, and this statistic would be denoted as the 3 DF test. The most significant SNPs were selected for further analyses of gene-smoking interaction effects.

We compare the results from 3 DF test with the homoscedasticity model. For homoscedasticity model, ever and never smokers were pooled and linear mixed model was fitted. We assume the same variances between never smokers and ever smokers, and the same coefficients of covariates as follows:

3 $$\begin{array}{rcl}{y}_{ij} & = & {\beta }_{0}+{\beta }_{1}{{\rm{age}}}_{i}+{\beta }_{2}{{\rm{sex}}}_{i}+{\beta }_{3}{{\rm{BMI}}}_{ij}+{\beta }_{4}{{\rm{height}}}_{ij}+{\beta }_{5}{{\rm{time}}}_{ij}+{\beta }_{6}{\rm{pack}}\,{{\rm{year}}}_{ij}\\  &  & +\,{\beta }_{7}{{\rm{sex}}}_{i}\cdot {{\rm{age}}}_{i}+\,{\beta }_{8}{\rm{smoking}}\,{{\rm{status}}}_{i}+{\beta }_{9}{{\rm{sex}}}_{i}\cdot {\rm{smoking}}\,{{\rm{status}}}_{i}\\  &  & +\,{\beta }_{10}{{\rm{SNP}}}_{i}+{\beta }_{11}{{\rm{SNP}}}_{i}\cdot {\rm{smoking}}\,{{\rm{status}}}_{i}+{\beta }_{12}{{\rm{SNP}}}_{i}\cdot {\rm{pack}}\,{{\rm{years}}}_{ij}\\  &  & +\,\sum _{k=1}^{10}{\tau }_{k}{{\rm{pc}}}_{i}^{k}+\,{\tau }_{11}{{\rm{pc}}}_{i}^{1}\cdot {\rm{smoking}}\,{{\rm{status}}}_{i}+{b}_{i}+{\varepsilon }_{ij},{({\varepsilon }_{ij1},\mathrm{...},{\varepsilon }_{i{n}_{i}})}^{t})\\  &  &  \sim MVN\,(0,{\rm{\Sigma }}),{b}_{i} \sim iid\,MVN(0,{\sigma }^{2})\end{array}$$

Then P-values for homoscedasticity model were obtained by likelihood-ratio tests with 3 degrees of freedom for H_0_: ${\beta }_{10}={\beta }_{11}={\beta }_{12}=0.$ This will be called homoscedasticity test in the remainder of this article.

#### Testing step: estimating the effects of SNPs and SNP-smoking interaction effects with KARE data

The 3 DF test provides P-values for overall effects about the main and interaction effects. However, it could not identify interaction effects of SNPs with smoking on lung function because never and ever smokers were separately analyzed. Furthermore, stratified analyses are usually less powerful compared to analyses using pooled data. Thus, former, current, and never smokers in KARE data were pooled and analyzed by a linear mixed model.

While building the linear mixed model, we first assessed variance-covariance structures by smoking status. If heteroscedasticity is not correctly taken into account, the false positive rates cannot be controlled with P-values^25^. Furthermore, modeling the relationship between smoking-related variables and FEV_1_ is not clear. If lung function of the participants worsens, they tend to quit smoking, and such indication biases make the relationships complicated. Thus, the appropriate choice of smoking-related variables may depend on sampling strategies. We considered various models with the choices of smoking-related variables and variance-covariance structure by smoking status for each dataset, and the best model was selected with the AICs. Notably, SNPs were not included for the model selection, and this step did not violate any statistical inference.

Supplementary Table S1 presents AICs for various models fitted to FEV_1_ for KARE data. Supplementary Table S1 shows that KARE data has the smallest AIC when smoking status had two levels (never versus ever smokers) instead of three levels, and different variances between never smokers and ever smokers were assumed. All covariates used for GWISs of KARE data were also included as covariates in our linear mixed model. We found that coefficients of some covariates differed according to smoking status, and interactions of some covariates and smoking-related variables were considered. For example, interactions between smoking status and time effects were significant at the 0.05 level and included as covariate. Furthermore, to control the effects of confounders on interaction effects, the interaction between confounders and environmental factors of interest should be considered for gene-environmental analyses^26^. Therefore, we considered interactions between PC scores for adjusting the population substructures and smoking status regardless of significance at 0.05 level. We considered *g* = 1 and 2 indicate ever smokers and never smokers, respectively, and *n*_*i*_ is the number of repeatedly observed measurements for participant *i*. The best model selected with AIC was as follows:

4 $$\begin{array}{rcl}{y}_{gij} & = & {\beta }_{0}+{\beta }_{1}{{\rm{age}}}_{i}+{\beta }_{2}{{\rm{sex}}}_{i}+{\beta }_{3}{{\rm{BMI}}}_{ij}+{\beta }_{4}{{\rm{height}}}_{ij}+{\beta }_{5}{{\rm{time}}}_{ij}\\  &  & +\,{\beta }_{6}{\rm{pack}}\,{{\rm{year}}}_{ij}+{\beta }_{7}{{\rm{sex}}}_{i}\cdot {{\rm{age}}}_{i}+\,{\beta }_{8}{\rm{smoking}}\,{{\rm{status}}}_{i}\\  &  & +\,{\beta }_{9}{{\rm{age}}}_{i}\cdot {\rm{smoking}}\,{{\rm{status}}}_{i}+{\beta }_{10}{{\rm{sex}}}_{i}\cdot {\rm{smoking}}\,{{\rm{status}}}_{i}\\  &  & +\,{\beta }_{11}{{\rm{height}}}_{ij}\cdot {\rm{smoking}}\,{{\rm{status}}}_{i}+{\beta }_{12}{{\rm{time}}}_{ij}\cdot {\rm{smoking}}\,{{\rm{status}}}_{i}\\  &  & +\,\sum _{k=1}^{10}{\tau }_{k}{{\rm{pc}}}_{i}^{k}+\,{\tau }_{11}{{\rm{pc}}}_{i}^{1}\cdot {\rm{smoking}}\,{{\rm{status}}}_{i}+{b}_{gi}\\  &  & +\,{\varepsilon }_{gij}\,,\,{({\varepsilon }_{gij1},\mathrm{...},{\varepsilon }_{gi{n}_{i}})}^{t} \sim MVN(0,{{\rm{\Sigma }}}_{g}),\,{b}_{gi} \sim iid\,MVN(0,{{\sigma }_{g}}^{2})\end{array}$$

This model includes two smoking-related variables—smoking status, and pack years. Therefore, both were used to define the interactions between SNP and smoking. To test gene and environment interactions, we considered

5 $$\begin{array}{rcl}{y}_{gij} & = & {\beta }_{0}+{\beta }_{1}{{\rm{age}}}_{gi}+{\beta }_{2}{{\rm{sex}}}_{gi}+{\beta }_{3}{{\rm{BMI}}}_{ij}+{\beta }_{4}{{\rm{height}}}_{gij}+{\beta }_{5}{{\rm{time}}}_{gij}+{\beta }_{6}{\rm{pack}}\,{{\rm{year}}}_{ij}\\  &  & +\,{\beta }_{7}{{\rm{sex}}}_{i}\cdot {{\rm{age}}}_{i}+{\beta }_{8}{\rm{smoking}}\,{{\rm{status}}}_{i}+{\beta }_{9}{{\rm{age}}}_{i}\cdot {\rm{smoking}}\,{{\rm{status}}}_{i}\\  &  & +\,{\beta }_{10}{{\rm{sex}}}_{i}\cdot {\rm{smoking}}\,{{\rm{status}}}_{i}+{\beta }_{11}{{\rm{height}}}_{ij}\cdot {\rm{smoking}}\,{{\rm{status}}}_{i}\\  &  & +\,{\beta }_{12}{{\rm{time}}}_{ij}\cdot {\rm{smoking}}\,{{\rm{status}}}_{i}+{\beta }_{13}{{\rm{SNP}}}_{i}+{\beta }_{14}{{\rm{SNP}}}_{i}\cdot {\rm{smoking}}\,{{\rm{status}}}_{i}\\  &  & +\,{\beta }_{15}{{\rm{SNP}}}_{i}\cdot {\rm{pack}}\,{{\rm{years}}}_{ij}{{\rm{s}}}_{i}+\sum _{k=1}^{10}{\tau }_{k}{{\rm{pc}}}_{i}^{k}+{\tau }_{11}{{\rm{pc}}}_{i}^{1}\cdot {\rm{smoking}}\,{{\rm{status}}}_{i}\\  &  & +\,{b}_{gi}+{\varepsilon }_{gij}\,,\,{({\varepsilon }_{gij1},\mathrm{...},{\varepsilon }_{gi{n}_{i}})}^{t} \sim MVN(0,{{\rm{\Sigma }}}_{g}),{b}_{gi} \sim iid\,MVN(0,{\sigma }_{g}^{2})\end{array}$$

#### Replication studies

SNPs selected from GWISs using KARE data were replicated with GENIE, MESA-Lung, and COPDGene data. For each dataset, we considered various variance-covariance structures, and the best model was selected with AICs. Supplementary Tables S2–S4 show AICs for GENIE, MESA-Lung, and COPDGene data, respectively, which were used as replication studies. The selected models with AICs were used to replicate the effects of SNPs and their interactions with smoking. Notably, SNPs and their interactions were not considered for model selection. A final model for replication data is described in Supplementary Text 2.

### Data availability

All data analyzed in this article were utilized in previously published articles (KARE: Cho, Go *et al*.^27^; GENIE: Choe, Lee *et al*.^20^; MESA-Lung: Hankinson, Kawt *et al*.^21^; COPDGene: Castaldi, Cho *et al*.^22^).

## Results

### Descriptive statistics

Table 1 shows baseline characteristics of participants in the KARE, GENIE, MESA-Lung, and COPDGene data. KARE and GENIE data were from the Korean population and included both baseline and longitudinal data. MESA-Lung and COPDGene were cross-sectional data, and participants in MESA-Lung were NHWs, whereas COPDGene data consisted of AAs and NHWs. In the KARE data, there were 8,534 participants, of which 47% were men. In the KARE data, participants were 40–69 years old, and the percentage of never smokers, former smokers, and current smokers were 58%, 20%, and 22%, respectively. The GENIE cohort composed of participants who were regularly screened for health, and their average lung function values were expected to be better than those of the general population. There were 5,971 participants, of which 57% were men. Participants were repeatedly measured an average of 3.13 times. In the GENIE data, participants were 30–84 years old, which explained the largest range of FEV_1_. In the GENIE data, the percentages of never smokers, former smokers, and current smokers were 56.9%, 30.2%, and 12.9%, respectively. MESA-Lung data consisted of 1,033 participants, of which 50.5% were men. These participants were 45–84 years old, and 44.4%, 45.3%, and 10.3% were never, former, and current smokers, respectively. Lastly, COPDGene data consisted of AAs and NHWs. The number of AAs was 3,300, of which 56% were men; these participants were 45–80 years old, and the percentages of former and current smokers were 20% and 80%, respectively. The number of NHWs was 6,670, of which 52% were men. Participants were 45–80 years old, and 61% and 39% were former and current smokers, respectively. Unlike KARE, GENIE, and MESA-Lung data, there were no never smokers in the COPDGene data, and participants in the COPDGene data had the lowest mean FEV_1_ and the highest pack years.

**Table 1:** Descriptive statistics Means of variables and their 95% confidence intervals are calculated.

	KARE	GENIE	MESA-Lung	COPDGene	
				AAs	NHWs
Participants	8534	5971	1033	3300	6670
Males/females	4001/4533	3404/2567	521/512	1846/1415	3493/3177
Age (years)	52.1 ± 17.4	47.1 ± 16.9	66.4 ± 19.2	54.7 ± 14.1	62.1 ± 17.2
Height (cm)	160.0 ± 17.1	166.4 ± 15.1	168.7 ± 18.8	171.2 ± 19.0	169.7 ± 18.6
Body mass index (kg/m^2^)	24.6 ± 6.1	23.1 ± 5.9	27.9 ± 10.2	29.1 ± 13.1	28.6 ± 12.0
Baseline FEV_1_ (liters)	2.9 ± 1.4	3.1 ± 1.3	2.5 ± 1.5	2.2 ± 1.7	2.1 ± 1.8
Baseline FVC (liters)	3.6 ± 1.8	3.8 ± 1.6	3.5 ± 1.9	3.1 ± 1.9	3.3 ± 2.2
Baseline FEV_1_/FVC (ratio)	0.8 ± 0.16	0.82 ± 0.14	0.73 ± 0.18	0.70 ± 0.25	0.63 ± 0.31
**Smoking status**					
never smokers	4926	3396	459		
former smokers	1742	1804	468	657	4054
current smokers	1866	771	106	2643	2616
Pack years	9.4 ± 31.4	6.7 ± 38.8	16.4 ± 51.2	38.3 ± 42.3	47.3 ± 51.0

### Heterogeneity of FEV_1_ decline along ages

**Figure 1:**
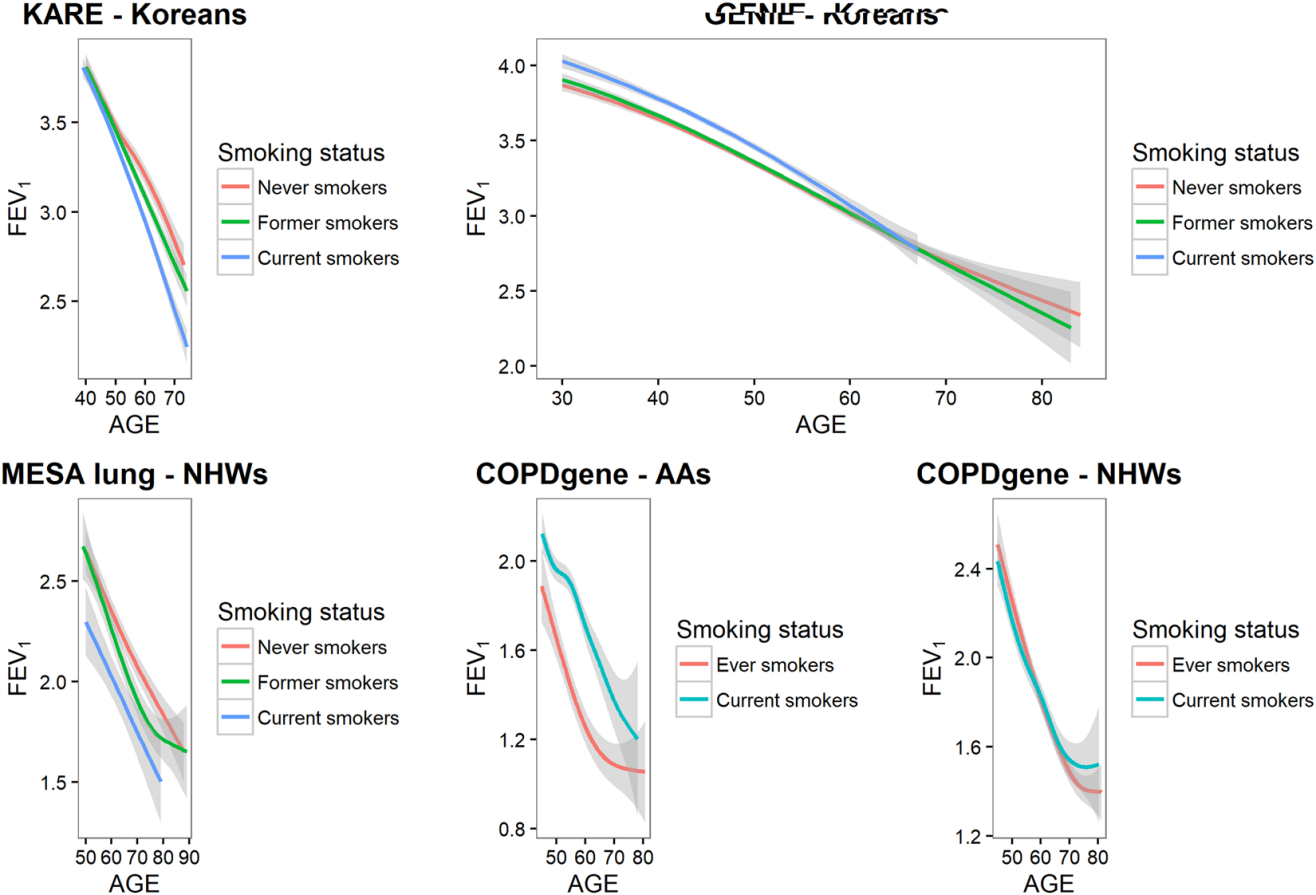
Changes of FEV_1_ along age Smoothing lines of FEV_1_ (L) according to age and their 95% confidence interval were estimated with generalized additive model.

Figure 1 shows the estimated FEV_1_ according to age and their 95% confidence intervals. The generalized additive models were applied for MESA-Lung and COPD gene data. KARE and GENIE data have the repeated measures of FEV_1_ levels, and the generalized additive mixed models were used. According to Figure 1, there were substantial differences among FEV_1_ according to the smoking status for each dataset. Current smokers in KARE and MESA-Lung data tended to have the lowest FEV_1_ values, followed by former smokers. For GENIE data, there were no differences in FEV_1_ values among never, former, and current smokers. Interestingly, for NHWs and AAs in COPDGene data, average FEV_1_ values of former smokers were smaller than those of current smokers, even though the differences were quite small for NHWs. This difference likely relates to selection bias, because participants with the worse lung function tended to quit smoking^28,29^, and only heavy smokers were considered for COPDGene. These results suggest that the same model could not be applied to different data to identify SNPs interacting with smoking, and the best model for the choice of smoking-related covariates and variance-covariance structures were selected with AICs.

### GWISs of FEV_1_ with KARE data

**Figure 2:**
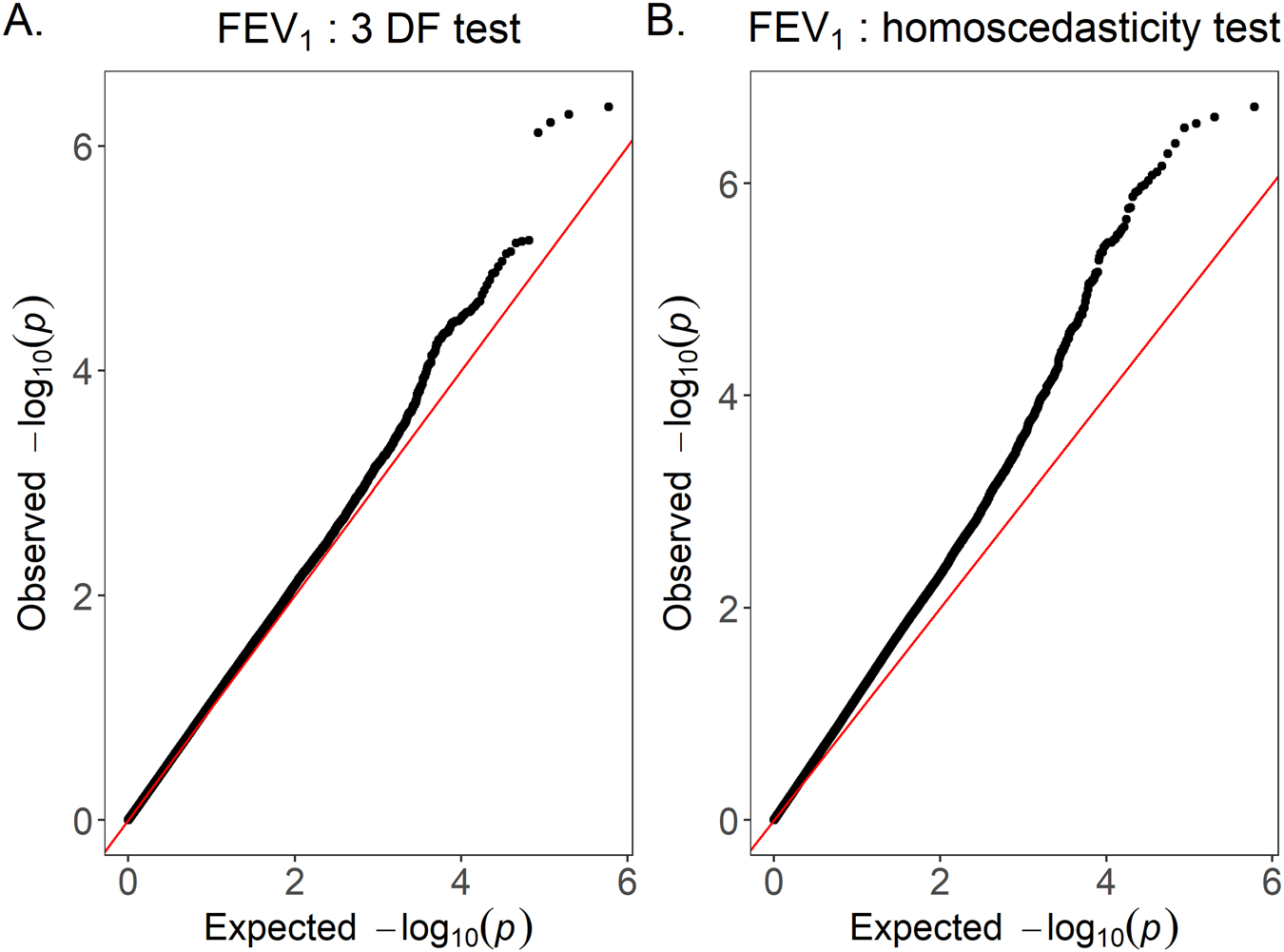
QQ plots for FEV_1_ Figure 2A is obtained from the proposed 3 DF test on FEV_1_ and Figure 2B is obtained from the homoscedasticity model.

In the discovery phase, associations of 310,515 SNPs were tested by applying the 3 DF test to KARE data. For the GWISs of FEV_1_, we included ten PC scores to adjust population substructure, and they explain 0.41% of genetic variances. Scatter plot for the first two PC scores and scree plot are provided in Supplementary Figure S1. Figure 2A presents the QQ-plot for 3 DF tests, and it shows that the proposed 3 DF tests generally preserve nominal significance levels. Variance-inflation factors (VIF) were estimated test was also applied^30^ and Figure 2B shows its QQ plot. Figure 2B reveals evidence of some inflation, and VIF of homoscedasticity model, 1.22, is substantially larger than 1. Supplementary Figure S2 is a Manhattan plot of results from the 3 DF test. We further checked the effects of heteroscedasticity according to smoking status on FEV_1_/FVC by applying the same methods, and there were no significant results (Supplementary Table S5). Supplementary Figure S3A,B are based on their results from 3 DF test and homoscedasticity test, respectively. VIFs for both models were 1.01 and 1.12. Therefore, we can conclude that heteroscedasticity according to smoking status should be carefully addressed for identifying the interaction effects between SNPs and smoking on lung function.

**Figure 3:**
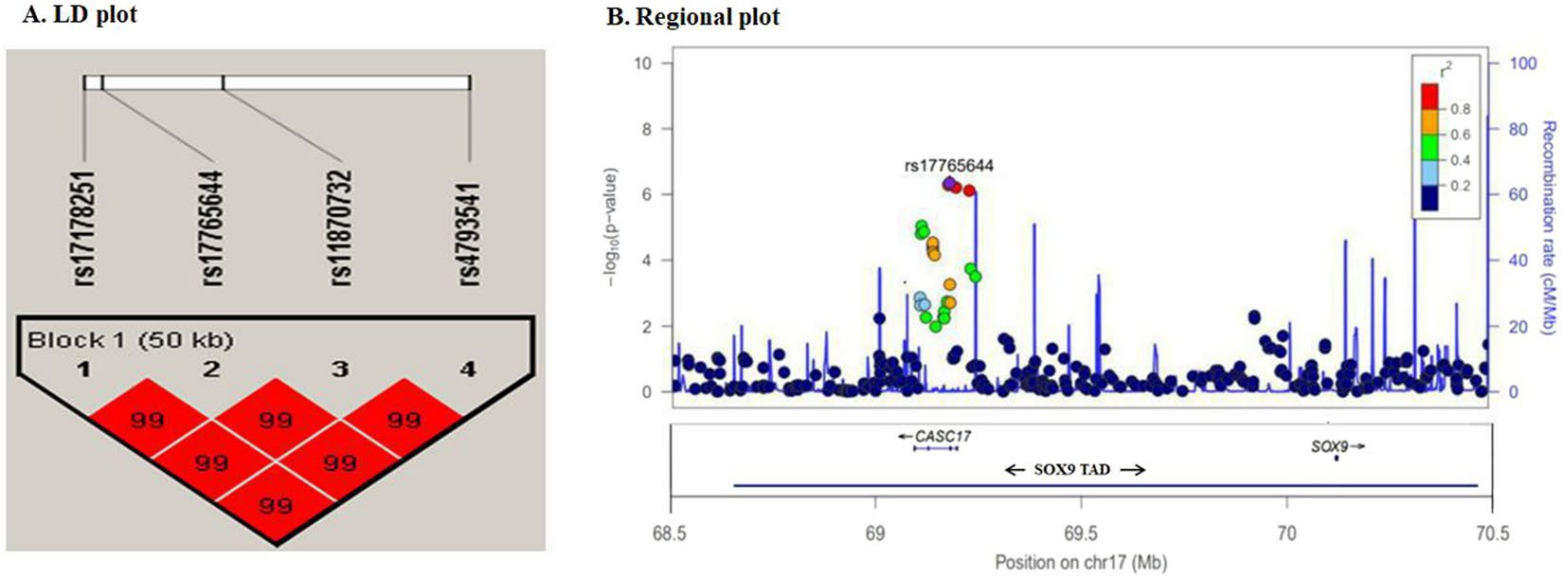
Linkage disequilibrium among the four most significant SNPs and regional plots Figure 3A shows the LD plot generated with Haploview software and D’ values were used. Figure 3B is a regional plot r^2^ around rs17765644 SNPs created with LocusZoom. *SOX9* TAD is located between 68.67 to 70.45 Mb.

Table 2 shows the most significant SNPs from results of 3 DF tests for FEV_1_. These selected SNPs were located in the upstream region of *SOX9* on chromosome 17 and had similar minor allele frequencies (MAFs). Furthermore the linkage distribution plot in Figure 3A reveals that these factors were highly correlated, which indicates that four significant SNPs actually indicate same association signal. Figure 3B shows the regional plot for this region. Those 4 SNPs are located within topologically associated domains (TAD) of *SOX9* region (68.67~70.45 Mb of chromosome 17 in hg19)^31–33^. DNA sequences within a TAD physically interact with each other more frequently than with sequences outside the TAD, and thus our most significant SNPs may affect the expression of *SOX9*. Therefore, our results indicate that *SOX9* may be functionally related with FEV_1_.

**Table 2:** Results from GWISs with KARE data 3 DF tests are conducted and the most significant SNPs were summarized.

SNP	Chromosome	Associated gene	Minor/Major alleles	MAFs	P-values for HWE test	P-values for 3 DF tests
rs17765644	17	*SOX9*	C/T	0.384	0.604	4.45 × 10^−7^
rs17178251	17	*SOX9*	G/C	0.383	0.572	5.21 × 10^−7^
rs11870732	17	*SOX9*	G/A	0.384	0.636	6.15 × 10^−7^
rs4793541	17	*SOX9*	C/T	0.391	0.324	7.63 × 10^−7^

-Definition of Abbreviations: SNP means single-nucleotide polymorphism, MAF means minor allele frequency, and HWE means Hardy-Weinberg equilibrium.

### Effects of SNPs and SNP-smoking interactions on spirometric measures in KARE data

**Figure 4:**
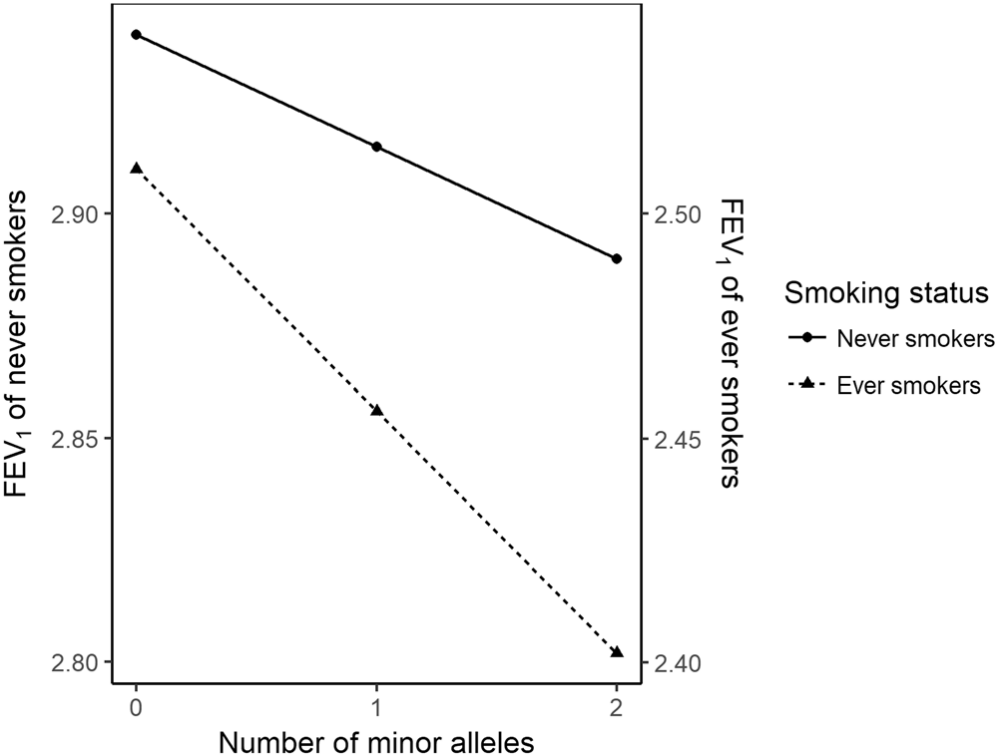
SNP-smoking interaction Figure 4 visualize the effects of rs17765644 by smoking status. X axis presents the number of minor alleles, and left and right of Y axis represent mean FEV_1_ values of never smokers, and ever smoker, respectively.

3 DF tests cannot separately estimate the main effects of SNPs and their interaction effects with smoking. In addition, ever and never smokers were separately analyzed, and such stratified analyses were less powerful than analyses with pooled data. Thus, all participants were pooled and analyzed with linear mixed models. Supplementary Table S1 shows AICs for the various models, and the selected model assumed different variances between never and ever smokers, corresponding to the linear mixed model eq. (5). This method was computationally very intensive and was applied to the most significant SNPs from GWISs. The selected models are summarized in Supplementary Table S6, and the Wald test of selected models for rs17765644, which has the smallest P-value in filtering step, are provided in Table 3. These results were obtained with PROC MIXED in SAS(version 9.4). The results for the other three SNPs are provided in Supplementary Tables 7–9 The results also showed that P-value for testing H_0_: ${\beta }_{{\rm{SNP}}}={\beta }_{\mathrm{SNP}-\mathrm{smoking}}={\beta }_{\mathrm{SNP}-\mathrm{PY}}=0$ was close to the Bonferroni-adjusted 0.05 significance level (1.61 × 10^−7^), and their SNP-smoking group interactions were significant at the 0.05 significance level, even though there were no significant interactions between SNPs and pack years. Coefficients for both the main association of rs17765644 and its interaction with smoking status were −0.025 and −0.029, respectively. This implies that the FEV_1_ of never smokers tend to be lower around (number of minor alleles) × 0.025 and ever-smokers are further lower around (number of minor alleles) × 0.029. Figure 4 shows the interacting effect of SNP with smoking status.

**Table 3:** Results for rs17765644 P-values for rs17765644 were obtained from the selected model for each data.

Data		Minor/Major alleles	MAF	HWE	Main effects	Interaction (SNP – smoking status)			Interaction (SNP – pack years)	Overall effects
					***β***_**SNP**_ (P-value)	never vs former *β*_SNP–SM1_ (P-value)	never vs current *β*_SNP–SM2_ (P-value)	former vs current *β*_SNP–SM3_ (P-value)	***β***_**SNP-PY**_ (P-value)	
Discovery	KARE (Koreans)	C/T	0.384	0.604	**−0.025 (2 × 10** ^**−4**^ **)**	**−0.029 (0.043)**			0.0004 (0.185)	**2.70 × 10** ^**−7**^
Replication	GENIE (Koreans)	C/T	0.380	0.164	−0.004 (0.336^*^)	−0.018 (0.052^*^)	**−0.024 (0.049** ^*^ **)**		0.0003 (0.981^*^)	**0.0820**
	MESA-Lung (NHWs)	C/T	0.438	0.521	**−0.064 (0.008** ^*^ **)**	0.078 (0.941^*^)			**−0.0021 (0.014** ^*^ **)**	**0.0037**
	COPDGene (AAs)	C/T	0.177	0.377	0.042 (0.499)			−0.097 (0.082)	0.0005 (0.555)	0.2205
	COPDGene (NHWs)	C/T	0.459	0.433	**−0.066 (0.020)**			0.049 (0.054)	0.0006 (0.200)	0.0746

*β*_SNP_ indicates the coefficient of the main effect of SNP. The smoking status was coded as dummy variables and never smokers were used as reference level. If three levels were defined, then two dummy variables are used. SM1 indicates the dummy variable which is coded as 1 for former smokers, and otherwise 0. SM2 indicates the dummy variable which is coded as 1 for current smoker and otherwise 0. SM3 is utilized only for COPDGene because there are no never smokers. 1 and 0 are for current and former smokers respectively. *β*_SNP–SM1_, *β*_SNP–SM2_ and *β*_SNP–SM3_ are the coefficients for the interaction between SNP and the corresponding dummy variables respectively. Since KARE and MESA-Lung data chose the smoking status with two levels (never vs ever smokers), *β*_SNP–SM1_ and *β*_SNP–SM2_ are shown. *β*_SNP–PY_ indicates the coefficient for the interaction between SNP and pack years. For GENIE, MESA-Lung data, we conducted one-tailed P-value based on the coefficients from KARE data and *indicates the results of one-tailed P-value. Overall effects indicate P-values for testing the null hypotheses ${\beta }_{{\rm{SNP}}}$ = ${\beta }_{\mathrm{SNP}-\mathrm{smoking}}$ = ${\beta }_{\mathrm{SNP}-\mathrm{PY}}$ = 0 by F test.

### Replication studies with GENIE, MESA-Lung, and COPDGene data

To validate the potential association of lung function with smoking, the four SNPs selected with KARE data were replicated in GENIE, MESA-Lung, and COPDGene data. According to the results from KARE data, the main effects of those four SNPs were negative, and their effects were more profound for ever smokers. Based on these factors, we conducted one-tailed tests for the main and interaction effects at the 0.05 significance level for replication studies.

Table 3 shows the results of replication studies for rs17765644. The best model for GENIE data was selected with AICs, and the selected model is shown in the eq. (1) of Supplementary Text 2 and Supplementary Table S6. According to Table 3, the main effect of rs17765644 was not significant for GENIE data. Smoking status had three levels, and two dummy variables were defined for GENIE data. Never smokers were used as the reference level, and P-value for overall test about the main effect of SNP, and interaction effects for SNP-smoking status, and SNP-pack years was significant at the 0.1 significance level. Interaction P-values between rs17765644 and dummy variables for former and current smokers were 0.052 and 0.049, respectively. The interaction effect between rs17765644 and dummy variables for current smokers was −0.024, which was much smaller than that between the SNP and dummy variable for former smokers. If former and current smokers were combined into ever smokers, and two levels were defined for smoking status, the estimated interaction effects between smoking status and rs17765644 and its P-values were −0.019 and 0.04, respectively.

The best model for MESA-Lung data was also selected with AICs, and the selected model is shown in the eq. (2) of Supplementary Text 2 and Supplementary Table S6. The smoking status for the best model has two levels, never and ever smokers. The dummy variable for smoking status is coded as 1 for ever smokers and as 0 for never smokers. As shown in Table 3, the P*-*value for overall effects was 0.0037. P-value for the main effect of rs17765644 was 0.008. The interaction effect between rs17765644 and smoking status was not significant, but its interaction with pack years was significant (P-value = 0.014). Thus, we concluded that the FEV_1_ values of ever and never smokers were not proportional to the coded genotypes, but the amount of decrease according to the pack years was proportional. Results for the other SNPs had a pattern similar to that of rs17765644 because they were highly correlated (Figure 3). These results are shown in Supplementary Tables 7–9

Lastly, AAs and NHWs in COPDGene data for rs17765644 were utilized to replicate the main effect and its interaction with smoking. For COPDGene data, there were no never smokers, and smoking status had two levels, former and current smokers. We found that former and current smokers had different FEV_1_ values, and the best model was selected with AIC. The selected models for AAs and NHWs are shown in the eqs (3) and (4) of Supplementary Text 2, respectively, and summarized in Supplementary Table S6. Former smokers were used as the reference level for smoking status. However, because there were no never smokers, and the directions of regression coefficients from the KARE data could not be considered, we used two-tailed tests. For AAs, the interaction effects of rs17765644 were significant at the 0.1 significance level (P-value = 0.082). Interestingly, rs11870732 (Supplementary Table S8) had significant interactions with smoking status (P-value = 0.037). For NHWs, the main effect of rs17765644 was significant at the 0.05 significance level, and the regression coefficient was −0.066, which was consistent with the data from other studies. However, its interaction effect with smoking status had the opposite direction (P-value = 0.054). This result may be attributable to the selection bias, and former smokers may have smoked more than the current smokers. The results for the other SNPs showed patterns similar to those of rs17765644.

## Discussion

We conducted GWISs of lung function (FEV_1_) to identify genetic variants interacting with cigarette smoking. We identified such an interaction using a joint test near the previously described *SOX9* locus on chromosome 17 in KARE. These findings were further explored in GENIE, MESA-Lung, and COPDGene. In this report, firstly, we replicated the main effects of *SOX9* on FEV_1_ values in Koreans, as shown in NHWs. Secondly, we found some evidence for *SOX9* gene-smoking interaction effects on FEV_1_, and former/current smokers with minor alleles of the selected SNPs near *SOX9* tended to have lower FEV_1_ values, even though the interaction effects were not strong. According to our results, the coefficient of gene-smoking interaction showed the same direction as the main SNP effect and its amount was almost the same. There have been very few studies showing a significant gene-smoking interaction effect, which could be replicated in other populations, though there were inconsistencies in the interaction effects for different ethnicities. Finally, we highlighted that the statistical model for the analysis of gene-smoking interactions should be carefully selected. The effects of smoking on FEV_1_ were very strong, and the means and variances could differ according to smoking status. Notably, when the mis-specified variance-covariance matrices were taken into account, QQ plots were inflated, and type-1 errors were not controlled.

*SOX9* has been extensively studied and shown to have pivotal roles in the lung epithelium during branching morphogenesis^17^. The epithelial-mesenchymal transition (EMT) is the process through which epithelial cells become mesenchymal-like, playing crucial roles in tissue repair and several pathological processes, including tissue fibrosis, tumor invasiveness, and metastasis. EMT is involved in specific steps in embryogenesis and organ development; however, this concept has been challenged by recent evidence showing that terminally differentiated epithelium can be changed to mesenchymal cells, even in adulthood^34^. This process can be activated by tissue injury or pathological stresses, and inappropriately controlled processes may induce tissue fibrosis and cancer. *SOX9* induces migratory fibroblasts responsible for extracellular matrix (ECM) deposition and tissue destruction by EMT^35^. *SOX9* induces lung fibrosis mediated by transforming growth factor (TGF)-β1 repair signaling, characterized by inappropriate ECM deposition; this can result in the destruction of tissue architecture and function^34,35^. Recent studies have suggested that *SOX9* activation is essential for the recovery of lung function after acute lung injury, and *SOX9* inhibition induces impaired recovery^18^. Smoking, which includes exposure to several oxidants and free radicals, causes numerous pulmonary diseases through inflammatory processes, leading to cell recruitment to the lung, activation of signaling pathways, and upregulation of proteins, consequently contributing to disruption of the lung ECM. This process varies from person to person; therefore, genetic susceptibility and gene-smoking interactions have been suggested to contribute to disease progression. Previous genome-wide joint meta-analyses of SNPs by smoking interactions on FEV_1_ and FEV_1_/FVC across 19 studies (total N = 50,047) demonstrated that *SOX9* was associated with FEV_1_ and was expressed at higher levels in the airway epithelium in smokers than in non-smoking adults of the European ancestry^19^. Our findings were consistent with the results of a previous study, and the *SOX9* gene-smoking interaction effects were verified in several ethnicities (Korean, NHWs, and AAs).

However, despite our interesting findings, some inconsistencies were observed in our replication results. For example, an interaction between smoking status and SNPs was found for KARE and GENIE data; however, for MESA-Lung data, the interaction between pack years and SNPs was significant. For COPDGene data, there were no never smokers, and former/current smokers were compared. Coefficients of smoking status and SNP interactions were significant at the 0.1 significance level, but had different directions for NHWs and AAs. There were multiple explanations for these phenomena, including ethnic differences^36,37^. Genetic ancestry itself is not assumed to be a cause for this difference, but could account for differences in lung function and susceptibility to smoking. For example, the structures of smoking experience vary by population^38^ implying complicated relationships between genetics, ethnicity, smoking, and lung function^39^. Therefore, the effects of gene-smoking interactions can be heterogeneous among different populations. In our replication study, we considered diverse ethnicities. According to our results, the replication with GENIE data was quite consistent with that of KARE data, and both were based on the Korean population. However, MESA-Lung and COPDGene data consisted of NHWs and AAs. Small differences between GENIE and KARE data could be explained by the characteristics of the participants. For example, KARE data were based on rural and urban community populations, and GENIE data were composed of participants who underwent regular health screening and received routine medical care. Medical care and routine health check-ups are often positively related to socioeconomic status^40^.

Furthermore, the effects of smoking on FEV_1_ are substantial; however, modeling its relationship with FEV_1_ is not clear. For example, we found that the effects of gene-smoking status were significant in the KARE and GENIE data, and in the MESA-Lung data, the effects of gene-pack years were significant. Smoking does not have a linear relationship with FEV_1_, and non-linear relationships^41,42^ have been reported frequently. The importance of various factors, such as smoking cessation time, smoking behavior, duration, and total dose, has been shown^43,44^ to explain the effects of smoking. In this report, we utilized the smoking status and pack years based on a self-reported smoking history. The prevalence of smoking from self-reporting surveys is usually underestimated, and the degree of underestimation varies among different countries^45–47^. To minimize this heterogeneity among different studies, we selected the best model with AIC. However, the appropriate definition for smoking is still unclear, and further studies are still necessary.

COPD is expected to be the third leading cause of mortality worldwide within a few years, and identifying genetic variants interacting with smoking would be beneficial in terms of social burden, aging, and the growing importance of personalized medicine^48^. However, statistical models that correctly model the effects of gene-smoking interactions based on lung function are complicated, and successful gene-smoking interaction analyses have been very limited. The proposed method illustrates the complexity of gene-smoking interaction analyses, and to identify consistent gene-smoking interactions, a statistical model should be developed that considers the non-linear relationships between smoking history and lung function.

## Electronic supplementary material

Supplementary Materials
