# Supplementary material for: Genome-wide assessment of gene-by-smoking interactions in COPD
Source: Sci Rep. 2018 Jun 18;8:9319. doi: 10.1038/s41598-018-27463-5 (PMC6006158; doi:10.1038/s41598-018-27463-5)
Supplement: Supplementary file 1 — Supplementary Materials [file 41598_2018_27463_MOESM1_ESM.pdf]

Web-based Supplementary Materials for "**Genome-wide assessment of gene-by-smoking interactions in COPD**"

Boram Park[1*], So-My Koo [2,3*], Jaehoon An[1], MoonGyu Lee[1], Hae Yeon Kang [4], Dandi Qiao[5], Michael H. Cho[5,6], Joohon Sung[1,7,8], Edwin K. Silverman[5,6], Hyeon-Jong Yang[3,9†], Sungho Won[1,7,8†]

[1]Department of public health science, Seoul national university, Seoul, Korea.

[2]Division of Allergy and Respiratory Medicine, Department of Internal Medicine, Soonchunhyang University Seoul Hospital, Soonchunhyang University College of Medicine, Seoul, Korea.

[3]SCH Biomedical Informatics Research Unit, Soonchunhyang University Seoul Hospital, Seoul, Korea

[4]Department of Internal Medicine, Healthcare Research Institute, Seoul National University Hospital Healthcare System Gangnam Center, Seoul, South Korea.

[5]Channing Division of Network Medicine, Department of Medicine, Brigham and Women's Hospital and Harvard Medical School, Boston, Massachusetts, United States of America.

[6]Division of Pulmonary and Critical Care Medicine, Department of Medicine, Brigham and Women's Hospital, Harvard Medical School, Boston, Massachusetts, United States of America.

[7]Interdisciplinary Program of Bioinformatics, Seoul National University, Seoul, Korea.

[8]Institute of Health and Environment, Seoul National University, Seoul, Korea.

[9]Pediatric Allergy and Respiratory Center, Department of Pediatrics, Soonchunhyang University Seoul Hospital, Soonchunhyang University College of Medicine, Seoul, Korea

[*]These authors contributed equally to this work and should be considered as co–first authors.

[†]These authors contributed equally to this work and should be considered as co–corresponding authors.

Corresponding Authors:

Sungho Won, Department of Public Health Science, Seoul National University

1 Kwanak-ro Kwanak-gu Seoul 151-742 Korea

(Email) won1@snu.ac.kr, (Tel) +82-2-880-2714, (Fax) +82-303-0942-2714

Hyeon Jong Yang, Department of Pediatrics, Soonchunhyang University Seoul Hospital,

Soonchunhyang University College of Medicine,

59 Daesagwan-ro, Yongsan-gu, Seoul, 04401, Korea

(E-mail) pedyang@schmc.ac.kr, (Tel) +82-2-709-9390, (Fax) +82-2-709-9083

Supplementary Text 1 Genotyping, quality-control, and imputation

KARE

Among 10,038 subjects, 10,004 were available, and they were genotyped with Affymetrix Genome-Wide Human SNP array 5.0[1]. For quality control (QC) tests, we excluded SNPs for which the missing genotype call rates were higher than 0.05, minor allele frequencies (MAFs) were less than 0.05, and Hardy-Weinberg equilibrium (HWE) P-values were less than 10^{-5}; additionally, participants with missing genotype call rates higher than 0.05 or with gender inconsistencies were excluded. After QC tests, 8,773 participants with 310,515 markers remained.

GENIE

The participants were genotyped using an Affymetrix customized chip[2]. Participants whose missing call rates were higher than 0.05 were excluded and conditions applied for SNP filtering were similar to those applied in KARE data. After QC tests, genotypes of 7,303 participants were included. Four SNPs selected from KARE data were not genotyped, and the imputed genotypes were utilized. The genotypes were first pre-phased using SHAPEIT2 and imputed with IMPUTE2. The 1,000 Genomes Phase 3 haplotypes was used as the reference panel, and the size of the buffer regions for imputations of target SNPs was set to 5 million base pairs. The estimated imputation accuracies for imputed SNPs, which are significantly associated to lung function in the discovery phase, were all higher than 0.9[3]. INFO for rs17765644, rs17178251, rs11870732, and rs4793541 was 0.99, 0.95, 0.93, and 0.99, respectively.

MESA-Lung

MESA-Lung study enrolled 3,965 participants who were sampled from the MESA cohort. We considered only NHWs, and they were genotyped using Affymetrix 6.0. The participants were excluded if the missing call rate was higher than 0.05[4] and 1,033 subjects were used for replication studies. Four SNPs selected from KARE data were genotyped and their call rates were all higher than 0.99. P-values of Hardy-Weinberg equilibrium tests for rs17765644, rs17178251, rs11870732, and rs4792541 were 0.52, 0.90, 0.86, and 0.61, respectively.

COPDgene

SNPs were genotyped using an Illumina Human Omni Express chip[5]. Participants were excluded[6] if their

missing call rates were higher than 0.01, relatedness by estimated identity-by-descent was higher than 0.125, or sex discrepancies or inbreeding coefficients were higher than 0.2. As a result, 7,760 NHWs and 3,300 AAs were utilized for replication studies[7]. Four SNPs selected from KARE data were not genotyped, and they were imputed with MaCH and minimac. The 1000 Genomes Phase v3 European and Cosmopolitan data were used as reference panels for NHWs and AAs, respectively. The minimac-RSQs[3] for rs17765644, rs17178251, rs11870732, and rs4793541 were higher than 0.98.

Supplementary Text 2 Final models for estimate the effects of SNP and SNP-smoking interaction in replication phase.

SNPs selected from GWISs with KARE data were replicated with GENIE, MESA-Lung, and COPDGene data. For each dataset, we considered various models associated with the choices of smoking-related covariates and variance-covariance structures, and models selected with AICs were used to replicate the effects of SNPs and their interactions with smoking. Notably, SNPs and their interactions were not considered for model selection. For GENIE data, smoking status with three levels—never/former/current smokers—was defined. Different means of FEV_1 were observed between former smokers and current smokers, and smoking status with three levels—never/former/current smokers—resulted in a better AIC. However, their variances were same. If we consider $g' = 1$ for never smokers, $g' = 2$ for former smokers, and $g' = 3$ for current smokers, the final model was as follows:

$$\begin{aligned}
y_{g'ij} &= \beta_0 + \beta_1 \text{age}_i + \beta_2 \text{sex}_i + \beta_3 \text{BMI}_{ij} + \beta_4 \text{height}_{ij} + \beta_5 \text{time}_{ij} + \beta_6 \text{pack year}_{ij} + \beta_7 \text{sex}_i \cdot \text{age}_i \\
&+ \beta_8 \text{smoking status}_i + \beta_9 \text{age}_i \cdot \text{smoking status}_i + \beta_{10} \text{sex}_i \cdot \text{smoking status}_i \\
&+ \beta_{11} \text{height}_i \cdot \text{smoking status}_i + \beta_{12} \text{time}_{ij} \cdot \text{smoking status}_i + \beta_{13} \text{SNP}_i + \beta_{14} \text{SNP}_i \cdot \text{smoking status}_i \\
&+ \beta_{15} \text{SNP}_i \cdot \text{pack years}_{ij} + \sum_{k=1}^{10} \tau_k \text{pc}_i^k + \tau_{11} \text{pc}_i^1 \cdot \text{smoking status}_i + b_{g'i} + \varepsilon_{g'ij}, \\
&(\varepsilon_{g'ij1}, \ldots, \varepsilon_{g'in_i})^t \sim MVN(0, \Sigma), \;\; b_{g'i} \sim iid\; MVN(0, \sigma^2) \cdots (1)
\end{aligned}$$

MESA-Lung data are cross-sectional data. If we consider $g = 1$ and 2 indicate ever smokers and never smokers, respectively, the selected model with AIC led to the following model:

$$\begin{aligned}
y_{gi} &= \beta_0 + \beta_1 \text{age}_i + \beta_2 \text{sex}_i + \beta_3 \text{BMI}_i + \beta_4 \text{height}_i + \beta_5 \text{pack year}_i + \beta_6 \text{sex}_i \cdot \text{age}_i + \beta_7 \text{smoking status}_i \\
&+ \beta_8 \text{age}_i \cdot \text{smoking status}_i + \beta_9 \text{sex}_i \cdot \text{smoking status}_i + \beta_{10} \text{sex}_i \cdot \text{age}_i \cdot \text{smoking status}_i \\
&+ \beta_{11} \text{SNP}_i + \beta_{12} \text{SNP}_i \cdot \text{smoking status}_i + \beta_{13} \text{SNP}_i \cdot \text{pack years}_i + \sum_{k=1}^{10} \tau_k \text{pc}_i^k + \tau_{11} \text{pc}_i^2 \cdot \text{smoking status}_i \\
&+ \varepsilon_{gi}, \; \varepsilon_{gi} \sim iid\; N(0, \sigma^2) \cdots (2)
\end{aligned}$$

Lastly, COPDGene data did not have any never smokers or fewer than 10 pack years smokers, and lung function differed between former smokers and current smokers. If we consider $g'' = 1$ for former smokers and 2 for current smokers, the considered model for AAs in COPDGene was

$$y_{g''i} = \beta_0 + \beta_1 \text{age}_i + \beta_2 \text{sex}_i + \beta_3 \text{BMI}_i + \beta_4 \text{height}_i + \beta_5 \text{pack year}_i + \beta_6 \text{sex}_i \cdot \text{age}_i + \beta_7 \text{smoking status}_i$$
$$+ \beta_8 \text{age}_i \cdot \text{smoking status}_i + \beta_9 \text{sex}_i \cdot \text{smoking status}_i + \beta_{10} \text{BMI}_i \cdot \text{smoking status}_i$$
$$+ \beta_{11} \text{pack year}_i \cdot \text{smoking status}_i + \beta_{12} \text{age}_i \cdot \text{sex}_i \cdot \text{smoking status}_i + \beta_{13} \text{SNP}_i$$
$$+ \beta_{14} \text{SNP}_i \cdot \text{smoking status}_i + \beta_{15} \text{SNP}_i \cdot \text{pack years}_i + \sum_{k=1}^{10} \tau_k \text{pc}_i^k + \tau_{11} \text{pc}_i^1 \cdot \text{smoking status}_i$$
$$+ \varepsilon_{g''i},\ \varepsilon_{g''i} \sim iid\ N(0, \sigma_{g''}{}^2) \cdots (3)$$

The final model for NHWs in COPDGene was as follows:

$$y_{g''i} = \beta_0 + \beta_1 \text{age}_i + \beta_2 \text{sex}_i + \beta_3 \text{BMI}_i + \beta_4 \text{height}_i + \beta_5 \text{pack year}_i + \beta_6 \text{sex}_i \cdot \text{age}_i + \beta_7 \text{smoking status}_i$$
$$+ \beta_8 \text{age}_i \cdot \text{pack year}_i + \beta_9 \text{smoking status}_i \cdot \text{pack year}_i \cdot + \beta_{10} \text{BMI}_i \cdot \text{pack year}_i + \beta_{11} \text{SNP}_i$$
$$+ \beta_{12} \text{SNP}_i \cdot \text{smoking status}_i + \beta_{13} \text{SNP}_i \cdot \text{pack years}_i + \sum_{k=1}^{10} \tau_k \text{pc}_i^k + \tau_{11} \text{pc}_i^1 \cdot \text{pack year}_i$$
$$+ \tau_{12} \text{pc}_i^4 \cdot \text{pack year}_i + \tau_{13} \text{pc}_i^5 \cdot \text{pack year}_i + \tau_{14} \text{pc}_i^6 \cdot \text{pack year}_i + \varepsilon_{g''i},\ \varepsilon_{g''i} \sim iid\ N(0, \sigma_{g''}{}^2) \cdots (4)$$

Supplementary Table 1 AICs for various models applied to KARE data AICs for different statistical models are calculated for KARE data. Smoking status can be never/former/current smokers or never/ever smokers. Ever-smokers indicates former or current smokers.

AIC	Structures of Σ_g and σ_g^2	Random effects structures of Σ_g	Correlation structures of Σ_g	Smoking status	Pack years
4,924.6	σ_g^2 = homo, Σ_g = homo	slope, intercept ; VC	VC	never/former/current	included
4,943.9	σ_g^2 = homo, Σ_g = homo	intercept	VC	never/ former /current	included
4,637.3	σ_g^2 = hetero, Σ_g = homo	intercept	VC	never/ former /current	included
4,592.9	σ_g^2 = hetero, Σ_g = hetero	intercept	AR	never/ former /current	included
4,961.7	σ_g^2 = homo, Σ_g = homo	slope, intercept ; VC	VC	never/ever	included
4,998.2	σ_g^2 =homo, Σ_g = homo	intercept	VC	never/ever	included
4,670.1	σ_g^2 = hetero, Σ_g = homo	intercept	VC	never/ever	included
8,852.8	σ_g^2 = homo, Σ_g = hetero	slope, intercept ; VC	VC	never/ever	included
4,379.2	σ_g^2 = hetero, Σ_g = hetero	slope, intercept ; VC	VC	never/ever	included
4,524.9	σ_g^2 = hetero, Σ_g = hetero	intercept	VC	never/ever	included
3,501.3	σ_g^2 = hetero, Σ_g = hetero	slope, intercept ; UN	UN	never/ever	included
**3,267**	**σ_g^2 = hetero, Σ_g = hetero**	**intercept**	**UN**	never/ever	**included**
3,706.6	σ_g^2 = hetero, Σ_g = hetero	intercept	UN	never/ever	excluded
4,374	σ_g^2 = hetero, Σ_g = hetero	slope, intercept ; UN	AR	never/ever	included
3,321.5	σ_g^2 = hetero, Σ_g = hetero	intercept	ARH	never/ever	included
3,621.6	σ_g^2 = hetero, Σ_g = hetero	intercept	ARH	never/ever	excluded
4,496.6	σ_g^2 = hetero, Σ_g = hetero	intercept	CS	never/ever	included

- Definition of Abbreviations: hetero assumes that Σ_g or σ_g^2 are different according smoking status, and homo assumes that Σ_g or σ_g^2 are same regardless of smoking status. VC means variance components correlation structure, UN means unstructured correlation structure, AR means Auto-regressive (1) correlation structure, ARH means heterogeneous AR correlation structure, and CS means compound symmetry correlation structure.

Supplementary Table 2 AICs for various models applied to GENIE AICs for different statistical models are calculated for GENIE data. Smoking status can be never/former/current smokers or never/ever smokers. Ever smokers indicates former or current smokers.

AIC	Structure of Σ_g and σ_g^2	Random effects structures of Σ_g	Correlation structure of σ_g^2	Smoking status	Pack years
-3,747,8	σ_g^2 = homo, Σ_g = homo	slope, intercept ; VC	VC	never/former/current	included
-3,654.2	σ_g^2 = homo, Σ_g = homo	intercept	VC	never/former/current	included
-2,960.2	σ_g^2 = hetero, Σ_g = homo	intercept	VC	never/former/current	included
-2,708.6	σ_g^2 = hetero, Σ_g = hetero	slope, intercept; VC	VC	never/former/current	included
-2,850.2	σ_g^2 = hetero, Σ_g = hetero	intercept	VC	never/former/current	included
-3,636.8	σ_g^2 = homo, Σ_g = homo	intercept	VC	never/ever	included
-3,437.4	σ_g^2 = hetero, Σ_g = homo	intercept; VC	VC	never/ever	included
6,233.7	σ_g^2 = homo, Σ_g = hetero	intercept	VC	never/ever	included
-3,640.7	σ_g^2 = hetero, Σ_g = hetero	slope, intercept; VC	VC	never/ever	included
-3,531.3	σ_g^2 = hetero, Σ_g = hetero	intercept	VC	never/ever	included
-3,623.2	σ_g^2 = homo, Σ_g = homo	slope, intercept ; UN	ARH	never/former/current	included
-3,57.68	σ_g^2 = homo, Σ_g = homo	slope, intercept ; VC	AR	never/former/current	included
-3,627.6	σ_g^2 = homo, Σ_g = homo	slope, intercept ; VC	CS	never/former/current	included
-3,521.7	σ_g^2 = homo, Σ_g = homo	slope, intercept ; VC	CS	never/former/current	excluded
**-3,934.1**	**σ_g^2 = homo, Σ_g = homo**	**intercept**	**AR**	**never/former/current**	**included**
-3,811.7	σ_g^2 = homo, Σ_g = homo	intercept	AR	never/former/current	excluded
-3,597.6	σ_g^2 = homo, Σ_g = homo	intercept	CS	never/former/current	included
-3,427	σ_g^2 = homo, Σ_g = homo	intercept	CSH	never/former/current	included

-Definition of Abbreviations: hetero assumes that Σ_g and σ_g^2 are different according to smoking status, and homo does that Σ_g and σ_g^2 are same regardless of smoking status. VC means variance components correlation structure, UN means unstructured correlation structure, AR means auto-regressive (1) correlation structure, ARH means heterogeneous AR correlation structure, and CS means compound symmetry correlation structure.

Supplementary Table 3 AICs for various models applied to MESA-Lung AICs for different statistical models are calculated for MESA-Lung data. Smoking status can be never/former/current smokers or never/ever smokers. Ever smokers indicates former or current smokers.

AIC	Structure of σ_g^2	Smoking status	Pack years
1,203.88	σ_g^2 = homo	never/ever	included
**1,170.08**	σ_g^2 **= hetero,**	**never/ever**	**included**
1,230.36	σ_g^2 = hetero	never/ever	excluded
1,203.88	σ_g^2 = hetero	never/former/current	included

-Definition of Abbreviations: hetero assumes that σ_g^2 are different according to smoking status, and homo does that σ_g^2 are same regardless of smoking status.

Supplementary Table 4 AICs for various models applied to COPDGene For COPDGene data, we consider the both of pack years and smoking status as interaction variables that could have interaction effects with age, sex, or height because COPDGene consists of only smokers.

(a) AAs in COPDGene

AIC	Structure of σ_g^2	Interaction variable	Pack years
6,022.78	σ_g^2 = homo	pack years	included
5,971.78	σ_g^2 = hetero	pack years	included
5,994.45	σ_g^2 = homo	smoking status	included
**5,940.65**	**σ_g^2 = hetero**	**smoking status**	**included**
5,954.54	σ_g^2 = hetero	smoking status	excluded

(b) NHWs in COPDGene

AIC	Structure of σ_g^2	Interaction variable	Pack years
14,504.34	σ_g^2 = homo	pack years	included
**14,488.41**	**σ_g^2 = hetero**	**pack years**	**included**
14,520.13	σ_g^2 = hetero	pack years	excluded
14,543.95	σ_g^2 = homo	smoking status	included
14,519.87	σ_g^2 = hetero	smoking status	included

-Definition of Abbreviations: hetero assumes that σ_g^2 are different according to smoking status, and homo does that σ_g^2 are same regardless of smoking status.

Supplementary Table 5 Results of FEV1/FVC from GWISs with KARE data 3 DF teste are conducted and the top 10 significant SNPs were summarized.

SNP	Chromosome	Physical Position	Associated gene	Minor/Major alleles	MAFs	P-values for HWE test	P-values for 3DF tests
rs7032628	9	101721984	COL15A1	A/C	0.3754	0.04512	4.41×10^{-7}
rs10512261	9	101724839	COL15A1	G/A	0.3185	0.3629	4.10×10^{-6}
rs10780572	9	85760746	-	G/A	0.1076	0.2909	4.52×10^{-6}
rs6826933	4	54445715	LNX1	T/C	0.2634	0.226	5.46×10^{-6}
rs1002824	4	54445980	LNX1	A/C	0.2626	0.5083	7.87×10^{-6}
rs10753780	1	168842901	-	T/A	0.4875	0.4675	1.03×10^{-5}
rs6702913	1	168837845	LOC105371606	T/C	0.4862	0.4807	1.04×10^{-5}
rs12464121	2	232379712	LINC00471	G/A	0.4708	0.8471	1.91×10^{-5}
rs9624873	22	25930784	-	T/A	0.1285	0.1524	2.78×10^{-5}
rs7427517	3	74146967	-	G/A	0.1276	0.2496	2.85×10^{-5}

Supplementary Table 6 Summary of selected models The best models selected with AIC are summarized for each data. Smoking status can be never/former/current smokers or never/ever smokers. Ever smokers indicates former or current smokers. Selected models for KARE, MESA-Lung, AAs in COPDGene, and NHWs in COPDGenes data assume heteroscedasticity variances according to smoking status and GENIE data chose homoscedasticity variance. All models included pack years.

Data	Study design	Smoking status
KARE (Koreans)	longitudinal	never and ever smokers
GENIE (Koreans)	longitudinal	never, former and current smokers
MESA-Lung (NHWs)	cross-sectional	never smokers and ever smokers
AAs in COPDGene (AAs)	cross-sectional	former smokers and current smokers
NHWs in COPDGene (NHWs)	cross-sectional	former smokers and current smokers

Supplementary Table 7 Results for rs17178251 P-values for rs17178251 were obtained from the selected model for each data. β_{SNP} indicates the coefficient of the main effect of SNP. The smoking status was coded as dummy variables and never smokers were used as reference level. If three levels were defined, then two dummy variables are used. SM1 indicates the dummy variable which is coded as 1 for former smokers, and otherwise 0. SM2 indicates the dummy variable which is coded as 1 for current smoker and otherwise 0. SM3 is utilized only for COPDGene because there are no never smokers. 1 and 0 are for current and former smokers respectively. $\beta_{\text{SNP-SM1}}$, $\beta_{\text{SNP-SM2}}$ and $\beta_{\text{SNP-SM3}}$ are the coefficients for the interaction between SNP and the corresponding dummy variables respectively. Since KARE and MESA-Lung data chose the smoking status with two levels (never vs ever smokers), $\beta_{\text{SNP-SM1}}$ and $\beta_{\text{SNP-SM2}}$ are shown. $\beta_{\text{SNP-PY}}$ indicates the coefficient for the interaction between SNP and pack years. For GENIE, MESA-Lung data, we conducted one-tailed P-value based on the coefficients from KARE data and * indicates the results of one-tailed P-value. Overall effects indicate P-values for testing the null hypotheses $\beta_{\text{SNP}} = \beta_{\text{SNP-smoking}} = \beta_{\text{SNP-PY}} = 0$ by F test.

	**Data**	**Minor/ Major alleles**	**MAF**	**HWE**	**Main effects**	**Interaction (SNP – smoking status)**			**Interaction (SNP – pack years)**	**Overall effects**
					**β_{SNP} (P-value)**	**never vs former $\beta_{\text{SNP-SM1}}$ (P-value)**	**never vs current $\beta_{\text{SNP-SM2}}$ (P-value)**	**former vs current $\beta_{\text{SNP-SM3}}$ (P-value)**	**$\beta_{\text{SNP-PY}}$ (P-value)**	
Discovery	KARE (Koreans)	G/C	0.383	0.604	**-0.025 (2 × 10^{-4})**	**-0.029 (0.046)**			0.0004 (0.178)	**3.28×10^{-7}**
Replication	GENIE (Koreans)	G/C	0.380	0.164	-0.003 (0.386*)	**-0.019 (0.049*)**	**-0.026 (0.038*)**		0.0003 (0.981*)	0.082
	MESA-Lung (NHWs)	G/C	0.432	0.521	**-0.060 (0.012*)**	0.064 (0.902*)			**-0.0016 (0.049*)**	**0.019**
	COPDGene (AAs)	G/C	0.173	0.377	0.043 (0.487)			-0.101 (0.073)	0.0005 (0.565)	0.189
	COPDGene (NHWs)	G/C	0.455	0.433	**-0.063 (0.028)**			0.043 (0.093)	0.0006 (0.227)	0.113

Supplementary Table 8 Results for rs11870732 P-values for rs11870732 were obtained from the selected model for each data. β_{SNP} indicates the coefficient of the main effect of SNP. The smoking status was coded as dummy variables and never smokers were used as reference level. If three levels were defined, then two dummy variables are used. SM1 indicates the dummy variable which is coded as 1 for former smokers, and otherwise 0. SM2 indicates the dummy variable which is coded as 1 for current smoker and otherwise 0. SM3 is utilized only for COPDGene because there are no never smokers. 1 and 0 are for current and former smokers respectively. $\beta_{\text{SNP-SM1}}$, $\beta_{\text{SNP-SM2}}$ and $\beta_{\text{SNP-SM3}}$ are the coefficients for the interaction between SNP and the corresponding dummy variables respectively. Since KARE and MESA-Lung data chose the smoking status with two levels (never vs ever smokers), $\beta_{\text{SNP-SM1}}$ and $\beta_{\text{SNP-SM2}}$ are shown. $\beta_{\text{SNP-PY}}$ indicates the coefficient for the interaction between SNP and pack years. For GENIE, MESA-Lung data, we conducted one-tailed P-value based on the coefficients from KARE data and * indicates the results of one-tailed P-value. Overall effects indicate P-values for testing the null hypotheses $\beta_{\text{SNP}} = \beta_{\text{SNP-smoking}} = \beta_{\text{SNP-PY}} = 0$ by F test.

	Data	Minor/ Major alleles	MAF	HWE	Main effects	Interaction (SNP – smoking status)			Interaction (SNP – pack years)	Overall effects
					β_{SNP} (P-value)	never vs former $\beta_{\text{SNP-SM1}}$ (P-value)	never vs current $\beta_{\text{SNP-SM2}}$ (P-value)	former vs current $\beta_{\text{SNP-SM3}}$ (P-value)	$\beta_{\text{SNP-PY}}$ (P-value)	
Discovery	KARE (Koreans)	G/A	0.384	0.636	**-0.025 (2 x 10^{-4})**	-0.028 (0.054)			0.0004 (0.174)	**3.95×10^{-7}**
Replication	GENIE (Koreans)	G/A	0.380	0.084	-0.001 (0.439*)	-0.018 (0.056*)	-0.024 (0.051*)		0.0003 (0.976*)	0.131
	MESA-Lung (NHWs)	G/A	0.431	0.864	**-0.060 (0.012*)**	0.062 (0.893*)			**-0.0016 (0.045*)**	**0.015**
	COPDGene (AAs)	G/A	0.162	0.254	0.079 (0.215)			**-0.120 (0.037)**	0.00009 (0.921)	0.151
	COPDGene (NHWs)	G/A	0.454	0.239	**-0.063 (0.026)**			0.042 (0.109)	0.0006 (0.216)	0.106

Supplementary Table 9 Results for rs4793541 P-values for rs4793541 were obtained from the selected model for each data. β_{SNP} indicates the coefficient of the main effect of SNP. The smoking status was coded as dummy variables and never smokers were used as reference level. If three levels were defined, then two dummy variables are used. SM1 indicates the dummy variable which is coded as 1 for former smokers, and otherwise 0. SM2 indicates the dummy variable which is coded as 1 for current smoker and otherwise 0. SM3 is utilized only for COPDGene because there are no never smokers. 1 and 0 are for current and former smokers respectively. $\beta_{\text{SNP-SM1}}$, $\beta_{\text{SNP-SM2}}$ and $\beta_{\text{SNP-SM3}}$ are the coefficients for the interaction between SNP and the corresponding dummy variables respectively. Since KARE and MESA-Lung data chose the smoking status with two levels (never vs ever smokers), $\beta_{\text{SNP-SM1}}$ and $\beta_{\text{SNP-SM2}}$ are shown. $\beta_{\text{SNP-PY}}$ indicates the coefficient for the interaction between SNP and pack years. For GENIE, MESA-Lung data, we conducted one-tailed P-value based on the coefficients from KARE data and * indicates the results of one-tailed P-value. Overall effects indicate P-values for testing the null hypotheses $\beta_{\text{SNP}} = \beta_{\text{SNP-smoking}} = \beta_{\text{SNP-PY}} = 0$ by F test.

	Data	Minor/ Major alleles	MAF	HWE	Main effects	Interaction (SNP – smoking status)			Interaction (SNP – pack years)	Overall effects
					**β_{SNP} (P-value)**	**never vs former $\beta_{\text{SNP-SM1}}$ (P-value)**	**never vs current $\beta_{\text{SNP-SM2}}$ (P-value)**	**former vs current $\beta_{\text{SNP-SM3}}$ (P-value)**	**$\beta_{\text{SNP-PY}}$ (P-value)**	
Discovery	KARE (Koreans)	C/T	0.391	0.324	**-0.024 (3 x 10^{-4})**	**-0.029 (0.044)**			0.0004 (0.174)	**5.44×10^{-7}**
Replication	GENIE (Koreans)	C/T	0.386	0.030	-0.003 (0.371*)	-0.018 (0.057*)	-0.022 (0.066*)		0.0003 (0.980*)	0.114
	MESA-Lung (NHWs)	C/T	0.450	0.610	**-0.065 (0.007*)**	0.072 (0.925*)			**-0.0018 (0.033*)**	**0.008**
	COPDGene (AAs)	C/T	0.271	0.862	-0.007 (0.893)			-0.013 (0.792)	0.00006 (0.441)	0.856
	COPDGene (NHWs)	C/T	0.473	0.625	-0.053 (0.060)			0.026 (0.318)	0.0005 (0.312)	0.234

Supplementary Figure 1 Principal component plots. Figure S1A is scatter plots of the first principal component and the second principal components. Figure S2B represents scree plot of eigenvalues.

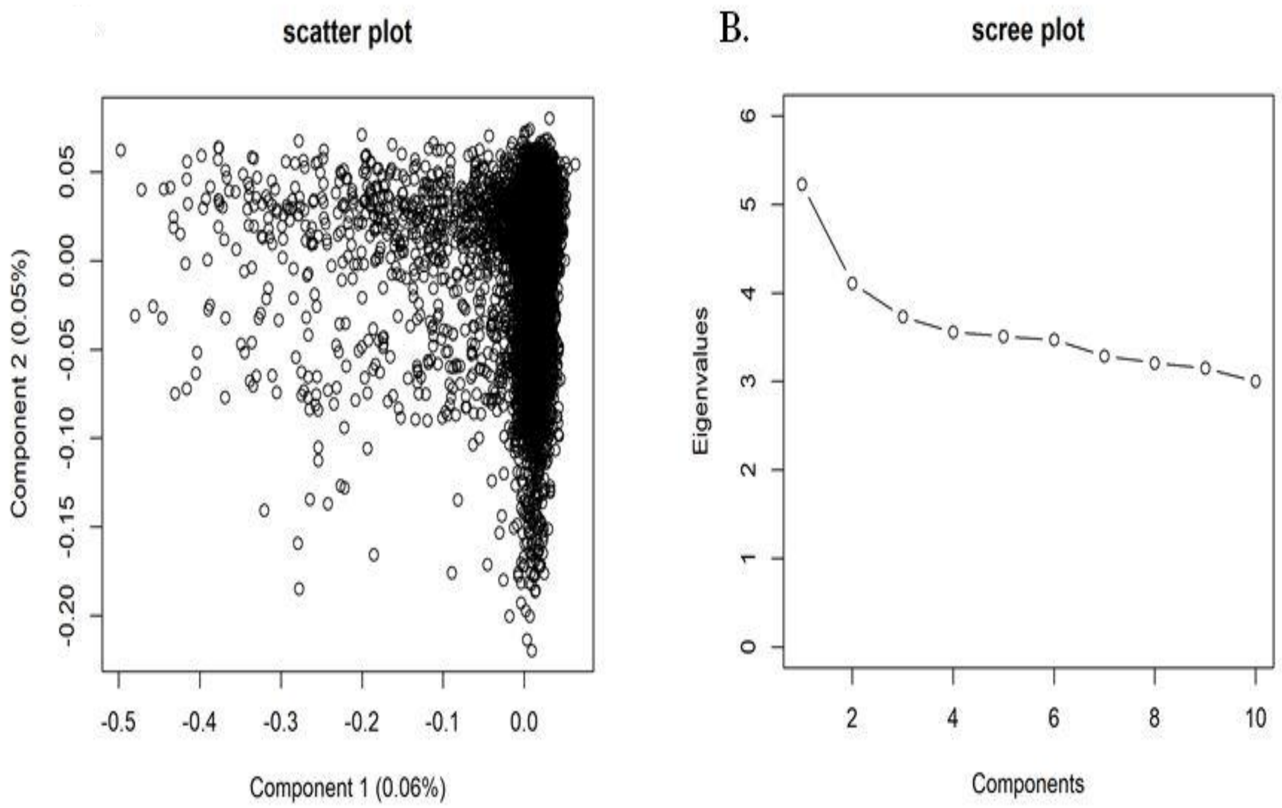

Supplementary Figure 2 Manhattan plot for FEV_1 Logarithms of the 3 DF P-values of 310,515 SNPs were plotted against its physical chromosomal position. The red and blue horizontal lines represent 1.71 x 10^{-7} (Bonferroni adjusted 0.05 significance level) and 10^{-6} respectively.

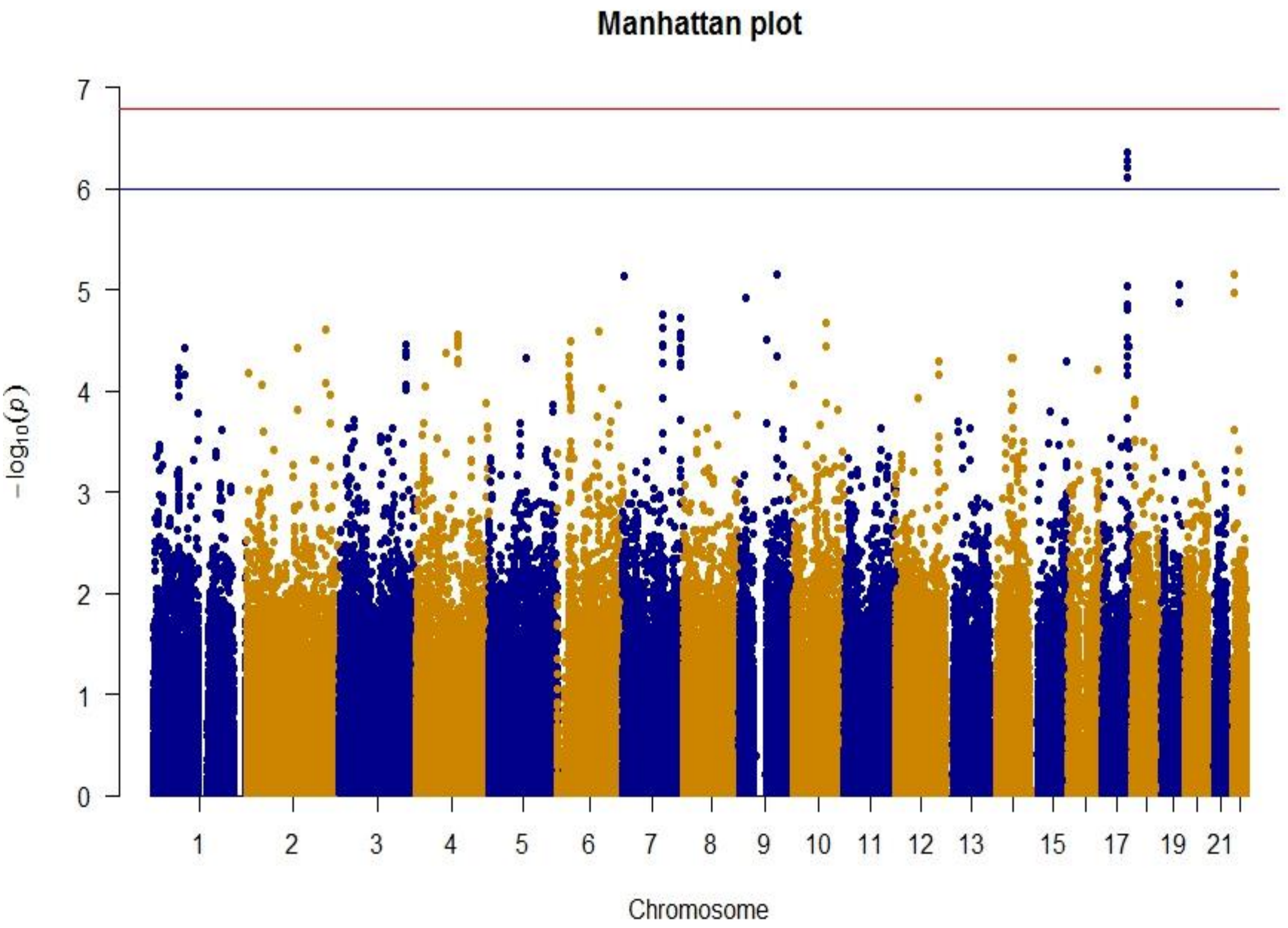

Supplementary Figure 3 Quantile-quantile plots for FEV_1/FVC Figure S3A is obtained from proposed 3 DF test on FEV_1/FVC and Figure S3B is obtained from homoscedasticity model by smoking status.

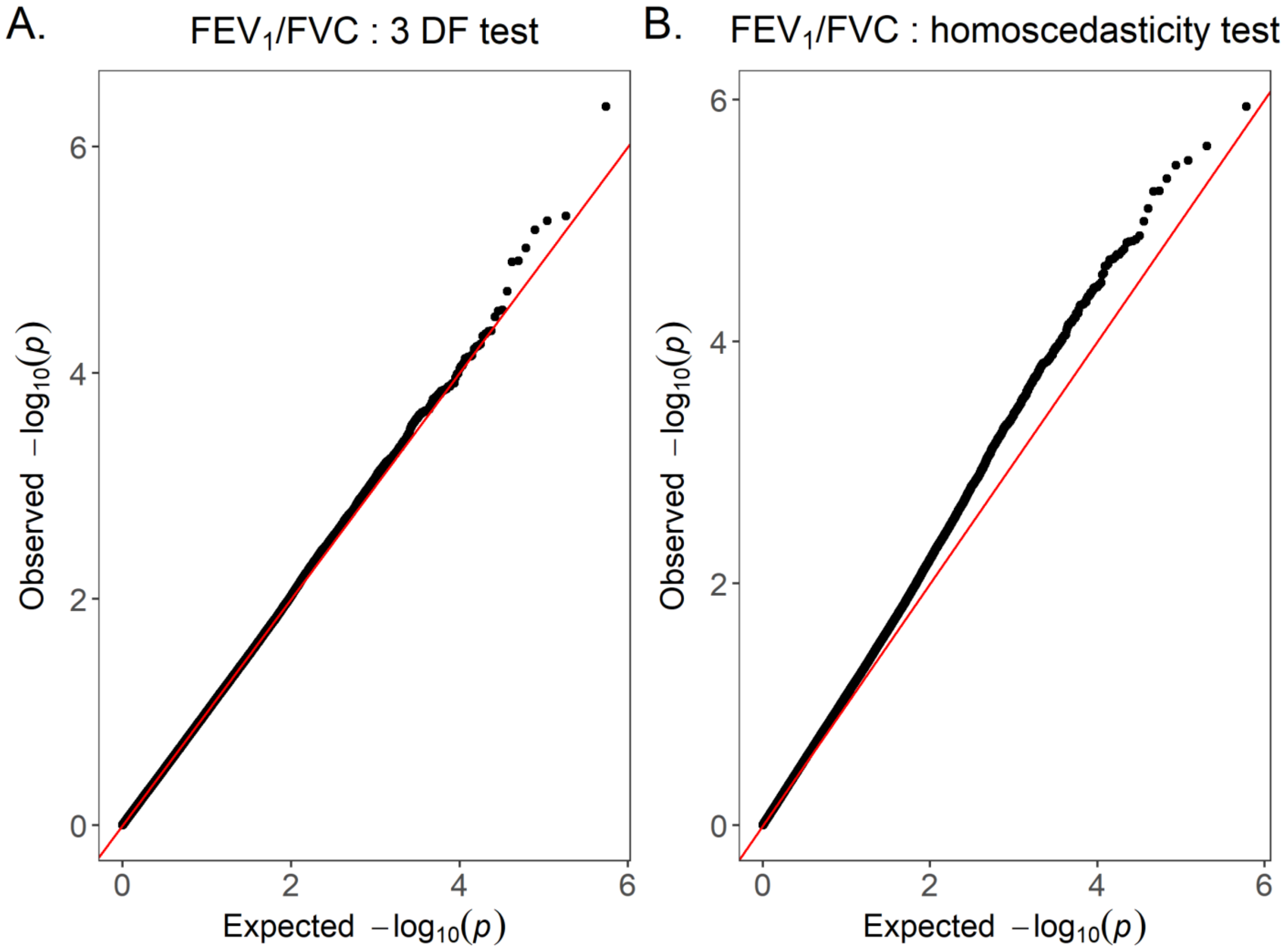

References

1 Cho, Y. S. *et al.* A large-scale genome-wide association study of Asian populations uncovers genetic factors influencing eight quantitative traits. *Nature genetics* **41**, 527-534 (2009).

2 Choe, E. K. *et al.* Search for genetic factor association with cancer-free prostate-specific antigen level elevation on the basis of a genome-wide association study in the Korean population. *European Journal of Cancer Prevention* (2017).

3 Gorski, M. *et al.* Harmonization of study and reference data by PhaseLift: saving time when imputing study data. *Genetic epidemiology* **38**, 381-388 (2014).

4 Manichaikul, A. *et al.* Association of SCARB1 Variants With Subclinical Atherosclerosis and Incident Cardiovascular Disease. *Arteriosclerosis, thrombosis, and vascular biology* **32**, 1991-1999 (2012).

5 Regan, E. A. *et al.* Genetic epidemiology of COPD (COPDGene) study design. *COPD: Journal of Chronic Obstructive Pulmonary Disease* **7**, 32-43 (2011).

6 Cho, M. H. *et al.* A genome-wide association study of COPD identifies a susceptibility locus on chromosome 19q13. *Human molecular genetics* **21**, 947-957 (2012).

7 Castaldi, P. J. *et al.* Genome-wide association identifies regulatory Loci associated with distinct local histogram emphysema patterns. *American journal of respiratory and critical care medicine* **190**, 399-409 (2014).
